# Sagittal Condylar Inclination and Transversal Condylar Inclination in Different Skeletal Classes

**DOI:** 10.3390/jcm11092664

**Published:** 2022-05-09

**Authors:** Anahit Lewandowska, Katarzyna Mańka-Malara, Jolanta Kostrzewa-Janicka

**Affiliations:** Department of Prosthodontics, Medical University of Warsaw, 02-097 Warsaw, Poland; anahit.lewandowska@wum.edu.pl (A.L.); jolanta.kostrzewa-janicka@wum.edu.pl (J.K.-J.)

**Keywords:** occlusion, Cadiax Compact, axiography, cephalometry, temporomandibular joint, temporomandibular disfunction, malocclusion, articulator, prosthodontics, orthodontics

## Abstract

Background: During prosthodontic treatment, the averaged values of the transversal condylar inclination (TCI) and the sagittal condylar inclination (SCI) are used for articulator settings. This study evaluated different parameters of measurable mandibular movements according to skeletal classes. Methods: Seventy-five patients (mean age 30.8 ± 5.49) had a condylography using the Cadiax Compact device (Gamma Dental, Klosterneuburg, Austria) and cephalometric analysis performed. Statistical analysis was performed using R statistical software. Results: There was no statistical evidence to state that the value of SCI angle is different in I compared to II Skeletal Class. There were no statistically significant differences when comparing the I vs. III and II vs. III Skeletal Class. The lowest mean SCI angle values were found in subjects with Skeletal Class III. There were statistically significant differences in left-sided TCI between Class I and II. There was a statistically significant linear relationship between ANB angle value and SCI value. Conclusions: Due to the wide individual variation in SCI and TCI values, it is advisable to use individual measurable parameters of mandibular movements during prosthetic reconstructions. The statistically significant relationship between SCI and ANB angle can be used to individualize the articulating parameters, especially in significant skeletal disproportions.

## 1. Introduction

Increasing expectations of modern life, stressful environments, and global turmoil lower our ability to adapt. Excessive mental loading has an impact on the stomatognathic system and can be a trigger for temporomandibular dysfunctions [1,2,3,4,5]. Additional local factors such as unbalanced occlusion favor the occurrence of such illness. Psychosocial stress is often accompanied by anxiety, hypervigilance, and somatosensory amplification contributing to pain [1]. Decreased compensatory abilities may also be caused by systemic diseases such as endocrine, skeletal, muscular, psychosomatic, and psychiatric disorders. When the indicated diseases are present, we may expect difficulties in achieving the clinical goals [6,7,8]. Moreover, all patients have a differing thin sensitivity to occlusal changes, which is influenced by many factors [9,10].

Prosthetic rehabilitation should not only replace missing tissues but also maintain the health of a patient [11,12,13]. Every dental appliance should be harmonized with the masticatory system, movement patterns, and even head posture. The use of appliances that track the movement of the mandible enables the calculation of the sagittal condylar inclination (SCI) and the transversal condylar inclination (TCI) [13,14,15,16]. Such devices are, for instance, the electronic Cadiax Compact (Gamma Dental, Klosterneuburg, Austria), ultrasonic ARCUSdigma (Kavo, Biberach, Germany) or optoelectric Condylocomp (Dentron, Höchberg, Germany). The use of the SCI, the TCI, the curve of Spee, the Wilson curve, and the inclination of the occlusal plane is necessary to individualize the prosthodontic treatment.

Current prosthodontics focus on novel materials mimicking nature. Digital treatment planning and 3D printing have brought us to a new era. In this era, however, we cannot ignore function and occlusion. Major skeletal and dentoalveolar discrepancies are corrected nowadays by orthognathic surgery and orthodontics. Many patients, however, have some skeletal disproportions camouflaged—by nature or an orthodontist—or only want to solve their dental problems by prosthodontic rehabilitation. Such treatment must always be conducted with respect to the patient’s anatomy. Therefore, the purpose of our research was to evaluate the differences between standard SCI and TCI parameters used in prosthodontics and those calculated according to skeletal classes.

## 2. Materials and Methods

The research included 75 patients (52 females and 23 males) aged from 22 to 44 years (mean 30.8 ± 5.49). They were recruited from among patients at the Medical University of Warsaw University Dental Center. All patients who met the inclusion criteria for the study and approved for participation were included in the research. The study protocol was approved by the Bioethics Committee at the Medical University of Warsaw (KB/189/2017). The investigation was carried out following the rules of the Declaration of Helsinki of 1975, revised in 2013. All participants have given written, informed consent to participate. They were also provided with a copy of the information along with their doctor’s contact information. The medical interview and dental examination included the evaluation of the stomatognathic system. The inclusion criteria included the presence of complete dentition and age between 18 and 45 years. The exclusion criteria were previous craniofacial surgery or orthodontic treatment, temporomandibular disorders, systemic diseases, and contraindications to radiography. A preliminary examination involved evaluating the mandibular range of motion and the soreness of the muscles of the head and neck region—the temporalis, masseter, lateral and medial pterygoid area, and sternocleidomastoid muscles.

**Figure 1:**
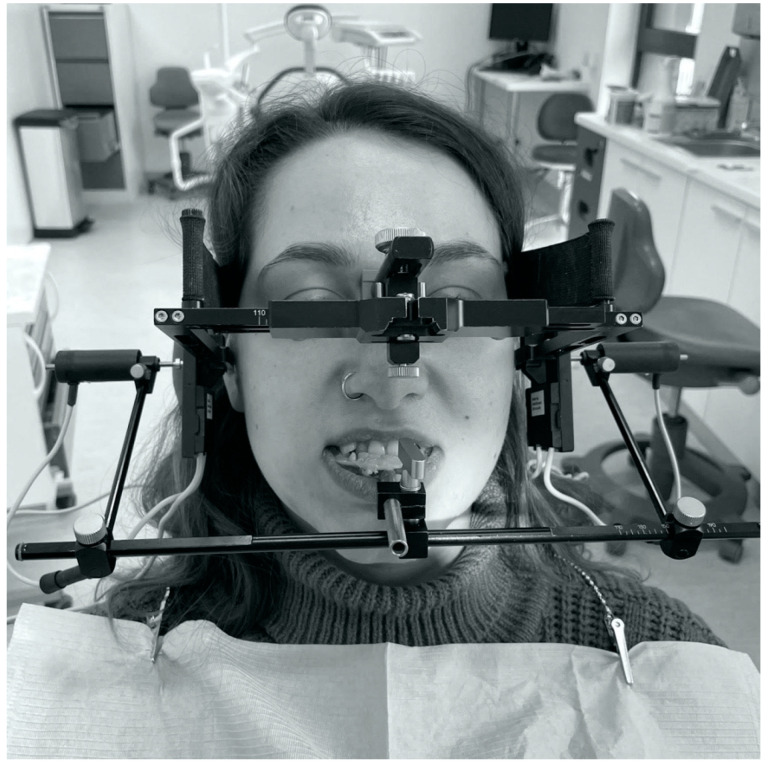
Condylography using the Cadiax Compact—front image.

**Figure 2:**
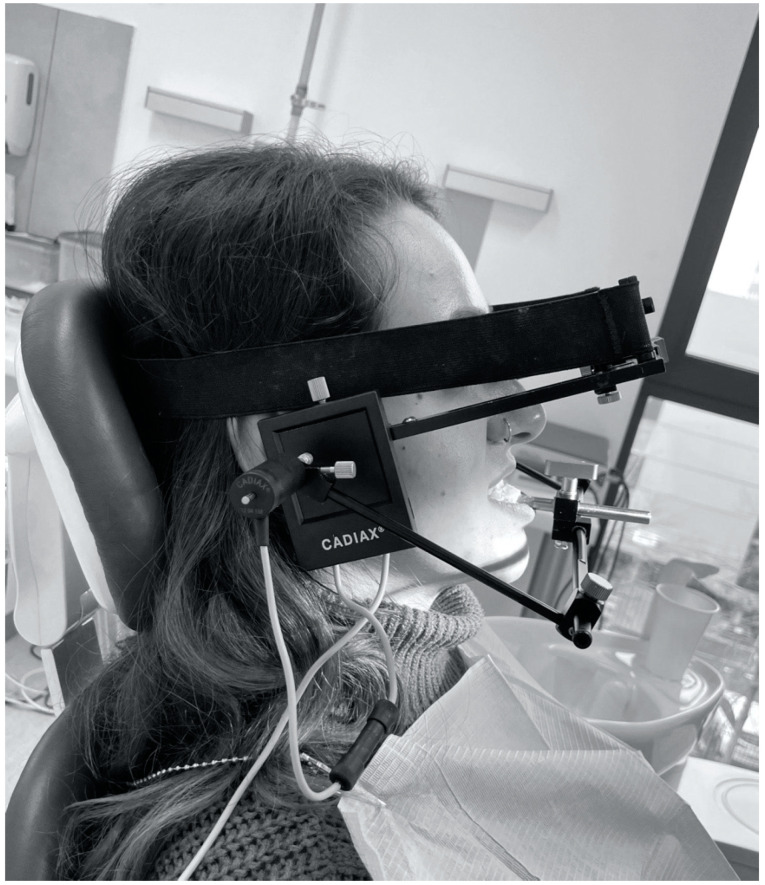
Condylography using the Cadiax Compact—lateral image.

**Figure 3:**
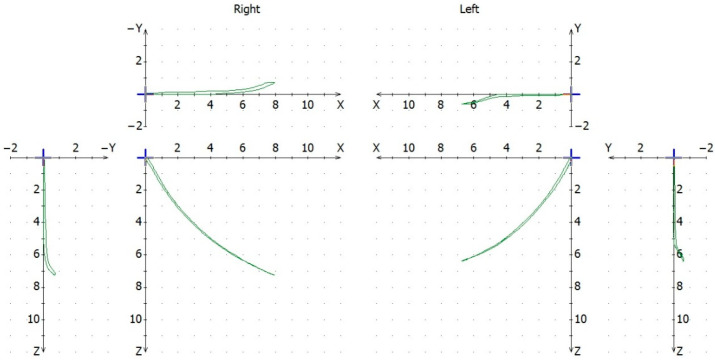
Registration in Cadiax Compact during the protrusion movement.

Condylography using the Cadiax Compact (Gamma Dental, Klosterneuburg, Austria) was used to obtain data for the articulator settings: the angle of the articular pathway in the sagittal and transversal planes (SCI, TCI) (Figure 1 and Figure 2). Patients practiced instructed mandibular movements before actual condylographic examination. The patient did maximal opening, protrusive, and lateral movements at a specified timing. Each practiced movement ended and began from the starting position (reference position)—the mandible located in the maximally retrusive position in which lateral movements were possible, without dental contacts and applied force. All movements of patients during the examination were carefully observed by the operator and registered in the software (Figure 3). The assessment was performed in the Gamma Dental software. SCI and TCI results obtained on 5 mm of motion were used for subsequent analysis. The cephalometric analysis was conducted for each participant of the study according to the Steiner analysis using the Gamma Dental software (Gamma Dental, Klosterneuburg, Austria). The sagittal jaw relationship was classified using the ANB angle. All condylographic registrations were done by one operator in the Department of Prosthodontics at the Medical University of Warsaw University Dental Center.

Statistical analysis was performed using the R statistical software [17]. Mean SCI and TCI values in the skeletal Class distributions were compared using the Student’s T-test (*p*-values and power analyses are reported in the next section). In each skeletal class, the SCI and TCI distributions showed the characteristics of a normal distribution according to the Shapiro-Wilk test. The relationship between the ANB angle, SCI, and TCI values were analyzed for the right and left temporomandibular joint (TMJ). Patients were divided into three groups: Skeletal Class I (23 patients), Skeletal Class II (29 patients), and Skeletal Class III (23 patients), according to the cephalometric analysis. 

To determine differences between measures within the skeletal classes, we conducted Student’s *t*-tests. The null hypothesis in Student’s *t*-test states that means within compared groups are equal, whereas we chose to pick a two-sided alternative hypothesis which states that means within compared groups are not equal. The assumed significance level (Type I error probability) was set to 0.05, where the statistical power (1 minus Type II error probability) for sample size calculations was set to 0.80. Type I error is the situation where the null hypothesis is true, but it’s rejected due to the analysis, whereas Type II error is the situation where the null hypothesis is not rejected while the alternative hypothesis is true. We calculated *p*-values (often treated as a probability of a null hypothesis is true) which is a boundary for specifying a statistically significant finding when compared to the significance level. Independently of *p*-values calculations, we also reported the statistical power [X] of each test. The statistical power of a hypothesis test is the probability of detecting an effect if there is a true effect present to detect (often explained as the probability that the test correctly rejects the null hypothesis. The sizes of each group (samples) have an impact on the outcome of the results of tests as their test statistics are based on sample means, sample standard deviations, and sample standard errors, which are influenced by the group (samples) sizes. Sample size calculations were reported as well to determine the minimum group size for the test to detect statistically significant findings for the observed sample means, sample standard deviations, sample standard errors, assumed Significance level (0.05), and assumed statistical power (0.80). Below we report the *p*-value, statistical power (power), and sample size needed to detect the effect (SSN) in parentheses [18].

## 3. Results

**Figure 4:**
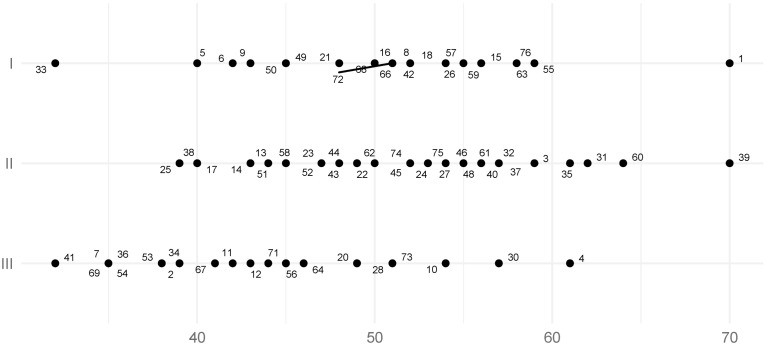
The distribution of the left sagittal condylar inclination (SCI) coefficient value. Numbers represent patients’ IDs.

The mean SCI value of the left TMJ for all studied groups was 49.2° (SD = 8.5°, SE = 0.98°), (Table 1), while the mean SCI value of the right joint for all studied groups was 49.7° (SD= 9.3°, SE = 1.07°) (Table 2). The result of the Student’s *t*-test for the SCI values of the left side in Skeletal Classes I and II did not provide sufficient grounds to conclude that the distributions between these classes were statistically significantly different (*p*-value = 0.69, power = 0.06, SSN = 1475). In Skeletal Class I, the mean left side SCI was 51° (SD= 7.90, SE = 1.65°), and in Skeletal Class II, the mean left side SCI was 51.8° (SD= 7.6°, SE = 1.41°). According to the results of the Student’s T-tests, these values were statistically significantly different from the SCI of the left TMJ for Skeletal Class III, for which the mean was 43.6° (SD= 8.1°, SE = 1.69°), (Table 1; Figure 4), (*p*-values: I vs. III = 0.005, II vs. III = 0.000; powers: I vs. III = 0.86, II vs. III = 0.95; SSNs: I vs. III = 19, II vs. III = 15)).

**Figure 5:**
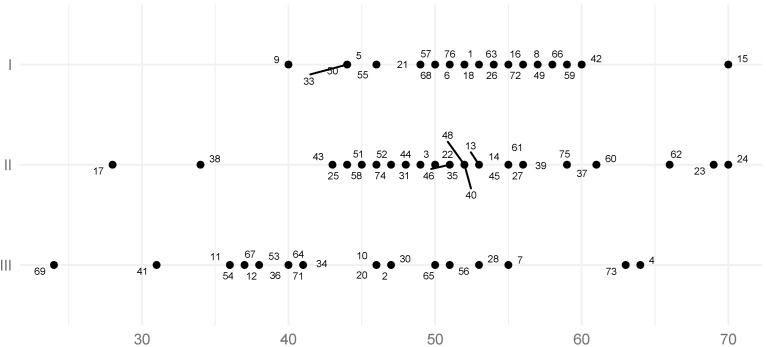
The distribution of the right sagittal condylar inclination (SCI) coefficient value. Numbers represent patients’ IDs.

There was no evidence to prove significant statistical differences between the SCI values for the right side when comparing Skeletal Classes I and II, according to the Student’s *T*-test (*p*-value = 0.85, power = 0.05, SSN ≥ 1000). In Skeletal Class I, the mean SCI of the joint was 52.2° (SD = 6.7°, SE = 1.40°), and in Class II it was 51.80 (SD = 9.2°, SE = 1.71°) (Table 2). According to the results of the Student’s *t*-test, these values were statistically significantly different from the SCI of the right joint in Skeletal Class III, which was 44.2° (SD= 9.70°, SE = 2.02°) (Figure 5), (*p*-values: I vs. III = 0.003, II vs. III = 0.008, powers: I vs. III = 0.88, II vs. III = 0.81, SSNs: I vs. III = 18, II vs. III = 25). It is worth noting that the SCI trends in the left and right joints according to skeletal classes were similar. For Skeletal Class II, the mean SCI values of the right and left sides were consistent.

**Figure 6:**
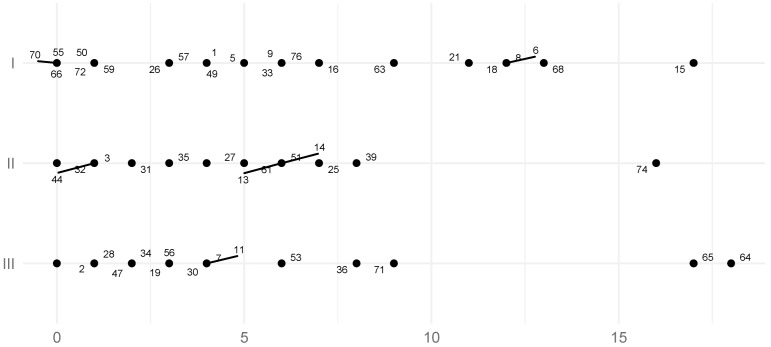
The distribution of the left transversal condylar inclination (TCI) coefficient value. Numbers represent patients’ IDs.

**Figure 7:**
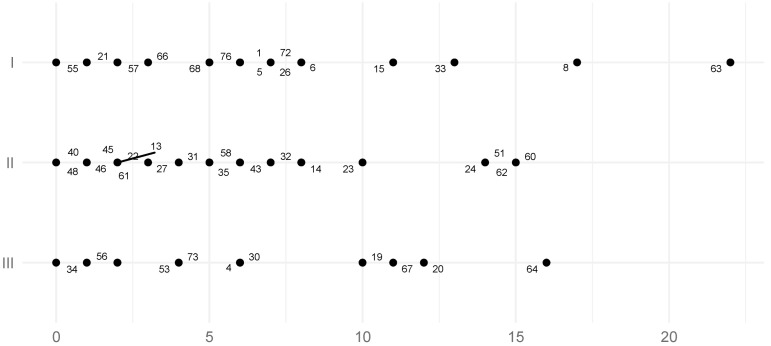
The distribution of the right TCI coefficient value. Numbers represent patients’ IDs.

The mean TCI of the left TMJ for all skeletal classes was 4.1° (SD = 4.6°, SE = 0.53°) (Table 3), while the mean TCI of the right TMJ for all study groups was 4.30 (SD = 5.20°, SE = 0.60°) (Table 4). According to the results of the Student’s *T*-tests, there was no significant statistical evidence to conclude that TCI values on the left side differed between Class II and III and Class I and III. The Student’s T-test determined that there were statistically significant differences in left-sided TCI between Class I and II. In Skeletal Class I the left side mean TCI was 6.0° (SD = 5.0°, SE = 1.04°), in Skeletal Class II 3.2° (SD = 3.6°, SE = 0.67°), while in Class III the mean was 3.6° (SD = 5.1°, SE = 1.06°) (Table 3; Figure 6), (*p*-values: I vs. III = 0.756, II vs. III = 0.107, I vs. II = 0.027, powers: I vs. III = 0.35, II vs. III = 0.06, I vs. II = 0.62, SSNs: I vs. III = 70, II vs. III ≥ 1000, I vs. II = 39). It could not be concluded that the TCI values of the right side in each skeletal class were statistically significantly different. In Skeletal Class I the right TMJ TCI was 5.2° (SD= 6.0°, SE = 1.250), in Skeletal Class II 4.1° (SD= 5.1°, SE = 0.95°), and in Skeletal Class III 3.6° (SD= 4.5°, SE = 0.94°) (Table 4; Figure 7), (*p*-values: I vs. III = 0.323, II vs. III = 0.692, I vs. II = 0.511, powers: I vs. III = 0.17, II vs. III = 0.06, I vs. II = 0.10, SSNs: I vs. III = 173, II vs. III ≥ 1000, I vs. II = 40).

In the second part of the analysis, the correlation between the right and the left side SCI and ANB angle was evaluated. For this purpose, in both cases, a linear regression model was fitted (Table 5). It was verified that the assumptions of both models were met and that the models’ quality of fits were appropriate (the residuals of the models had normal distributions, and the variances of the residuals were homogeneous). The models showed mean values of left SCI (47.5) and right SCI (48.5) and statistically significant coefficients for ANB: left SCI (0.77) and right SCI (0.72). This means that (according to linear models), on average, the left SCI was 47.5, and for each ANB, it should be increased by ANB × 0.77, and on average, the right SCI was 48.5, and for each ANB, it should be increased by ANB × 0.72. In the model for the left SCI, the result of the general F-test for the linear model stated that this model was statistically significantly more effective in predicting left SCI based on ANB than when predicting left SCI without any knowledge (null model) (*p*-value = 0.012). The R^2^ statistic was 0.098, meaning that ANB explains 9.8% of the variability associated with the left SCI distribution. For the model predicting right SCI, the general F-test for the linear model shows that the model is statistically significantly more effective in predicting right SCI based on ANB than when predicting right SCI without any knowledge (null model) (*p*-value = 0.039). The R^2^ statistic was 0.069, meaning that ANB explains 6.9% of the variability associated with the right SCI distribution. 

## 4. Discussion

The use of individual TMJ movement parameters is important to provide properly fitted prosthetic restorations. The setting of SCI and TCI values in articulators is an important factor in the individualization of occlusal reconstruction. In the obtained results, the mean SCI value calculated for the left TMJ was 49.2°. The same value for the right joint was 49.7°. The average SCI, according to literature, is between 20–33° [19]. These findings are comparable with studies by other authors. 

Using the Cadiax Compact, Ruwaida et al. [20] showed a mean SCI for the left joint that was 41.9° (SD = 9.2°) and for the right joint 42.0° (SD 8.5°). This study showed no statistically significant differences in SCI values for the left and right TMJs between Skeletal Class I and II subjects. In the left joint, the correlation of SCI in Skeletal Class I compared to Skeletal Class II was 51° vs. 51.8°. Statistically significant different (*p* < 0.05) results were presented by Canning et al. [21], who obtained SCI values for the left joint in Skeletal Class I of 46.38° compared to Skeletal Class II of 48.93°. Similar correlations were found in the evaluation of the right joint SCI in Skeletal Class I vs. Skeletal Class II 44.13° vs. 49.00°. Statistical analysis was performed with the Student’s *t*-test, and SCI values were obtained with the Cadiax Compact device, like in the presented study. However, the classification of classes in that research was obtained by evaluating profile photographs of the patients. Another difference between the study conducted by the authors Canning et al. [21] is that no statistical significance was found between Class I and III, whereas in the results obtained in our study, statistically significant differences were found between Skeletal Class I vs. III (*p* = 0.005 in the left joint, *p* = 0.003 in the right joint), and Skeletal Class II vs. III (*p* = 0.000 in the left joint, *p* = 0.008 in the right joint). The obtained SCI values in Skeletal Class III are the lowest among all researched groups in both studies. Zimmer et al. [22] compared SCI values in skeletal classes similarly to the aforementioned study and obtained significant statistical differences between Skeletal Classes I and III. This study also shows the highest SCI values in Skeletal Class II, which is not reported in our study, but is consistent with the results of Canning et al. [21].

In presented research, no statistically significant differences in TCI values were found between I vs. III and II vs. III skeletal classes in the left joint. Statistical significance of the difference in TCI values occurred only in the comparison of I vs. II Skeletal Class in the left joint. Cimić et al. [23] found no statistically significant relationship between the TCI value and the Angle Class exhibited in the results of their study. The average TCI value in our study was 4.1° in the left joint and 4.3° in the right joint. In the study by Cimić et al. [23] the value was 6.3° in the left joint and 7.7° in the right joint. The highest TCI values were found by the authors in subjects with Angle class III, which is not consistent with the results of the abovementioned study. However, the Angle classification reports only the dentoalveolar discrepancies, while in current research, the compared patients were classified in sagittal skeletal classes based on ANB angle. The used parameter may influence the achieved results as the studies on the validity of the ANB and Wits appraisal to show that there are many distorting factors. The ANB angle can differ because of variance in the length of the cranial base or rotation of the jaws [24,25,26,27], while the Wits appraisal can be affected by the inclination of the occlusal plane [28,29,30].

The highest TCI value was obtained in Skeletal Class I. Mean TCI values in this study in all skeletal classes are also different from the results of other authors who obtained higher values of the above parameters [31]. These differences may be related to different research protocols and methodology. The use of measuring devices to determine TCI gives, on average, results with lower values of these parameters compared to the measurement performed with occlusal registrations [32,33,34,35]. At the same time, all obtained average TCI results in the mentioned articles are lower than those routinely used for articulator settings—amounting to 15° [23]. Obtained results show a significant statistical relationship between SCI and ANB. The ANB angle value explains 9.8% of the variability associated with the left SCI distribution and 6.9% of the variability associated with the right SCI distribution. This means that there is a linear relationship between ANB and SCI. At the same time, the value of the SCI angle is influenced by other modifying factors. The influence of various factors on SCI has been studied in the literature, but often the results were not statistically significant. Among others, the impact of age, gender, condylar process shape, missing teeth, and TMJ disorders (symptoms and signs of TMD) on the influence of the parameters of measurable mandibular movements were negated [21,33,34]. The SCI and TCI parameters in various skeletal patterns are clinically comparable. It would not be a major mistake to use standardized values for all skeletal classes. However, in the current study, we presented the recommended corrections for SCI according to the ANB angle. The introduction of such a formula is clinically important. However, the suggested corrections should be verified in further studies on larger groups.

In the presented research, the SCI and TCI results were obtained on 5 mm of motion. The reason for choosing that parameter was to have a relationship in which we could achieve the necessary space for possible prosthodontic reconstruction and to avoid possible occlusal interferences caused by a malocclusion. There are publications in the literature that did not consider the value of the displacement path in protrusion motion in the determination of SCI values [35,36,37,38,39,40]. Studies taking into account the value of the displacement path in protrusion motion showed that there are no statistically significant differences in the calculation of SCI values depending on measurements made on 3 mm or 5 mm of movement in the protrusion (41.22 ± 9.71 degrees vs. 41.89 ± 8.62 degrees). The authors also recommend recording the SCI at 5 mm of protrusion because it provides a more accurate interpretation of the condylar process motion that is not affected by the natural curvature of the condylar path that occurs at the first 3 mm of motion. There were also no statistically significant differences between the SCI value calculated at 3 mm and 5 mm when comparing the Frankfurt and Camper reference planes [16,34,35,36,41,42,43]. There were statistically significant differences in SCI values at 3 mm and at 5 mm in studies using either the Frankfurt or Camper compared to the AOP plane. Studies using the Frankfurt plane were conducted by Theusner et al. [44] using the SAM electronic device (mean SCI 35 ± 7 degrees) and by Han et al. [42] using the CADIAX electronic device (mean SCI 40.01 ± 8.12) on the Frankfurt plane show statistically significant differences—approximately 15 degrees smaller values—compared to studies conducted using the Axis Orbital Plane (AOP) on SAM axiography by Kucukkeles et al. [37] (mean SCI 53.3) or Boulos et al. [45] (mean SCI 51.4 ± 9.75 degrees).

There are many publications in the current literature on differences resulting from various skeletal patterns. Patients with skeletal malocclusion have impaired masticatory abilities and performance in comparison with the control group [34,46]. English et al. [46] also found that individuals with Class III malocclusion had the lowest masticatory performance compared with other malocclusion groups. However, in this research, vertical relations were not considered. Research conducted by Ugoloni et al. [47], which analyzed nine Skeletal Class III patients scheduled for orthognathic surgery, showed that the range of movement in Class III patients was comparable to that found in normal subjects. They stated that TMJ kinematics in such subjects was modified, both left and right condyles had a variable degree of hypomobility, and the condyle translational movement was reduced. Abrahamsson et al. [34] described that open bite is the discrepancy that has an impact on the masticatory performance index (MPI). They suggest that the increase in occlusal contacts after orthodontic treatment may contribute to higher efficiency in mastication. Tamimi et al. [48] stated that occlusal bite force greatly improves after surgical correction of vertical morphology in high angle mandibular prognathic patients. The bite force is correlated with parameters such factors as the mandibular length and mandibular angle at the gonion point, facial length, and the masseter muscle thickness [49,50,51]. Kostrzewa-Janicka [52] described a formula, calculated by cephalometric analysis, for a vertical jaw separation in which the bite force is minimal.

Many authors also concentrate on comparing TMJ anatomy in different skeletal relationships. Santander et al. [53] show a correlation between skeletal patterns and condylar morphology in the adult population. They conducted a CBCT study on 111 patients showing that Skeletal Class II correlates with smaller, shorter, and more inclined condyles compared to Class III subjects. Facial asymmetry determined by menton point deviation did not mirror differences in condylar shape or inclination. Similar results were achieved by Hasebe et al. [54], who described that Class II patients or those with hyperdivergent skeletal patterns had small condylar sizes, and subjects with Class III or hypodivergent skeletal patterns had large condylar sizes. Additionally, females had smaller condyles than males. There are also differences in the condyle-fossa relationships between patients with different skeletal patterns [55,56,57]. Despite reported differences, neither the sex nor the dimension of the condyle influence the SCI. The condyle is positioned more inferiorly in hypodivergent than hyperdivergent Skeletal Class II patients. According to Katsavrias [58], the articular eminence height and articular eminence inclination are highly correlated to ramus inclination.

The major limitation of the presented study is the small sample size for the variables in the assessment. However, data published by other authors using similar methodology included comparable or smaller groups. Han et al. [42] conducted research on a group of 10 patients, Torabi et al. [16] included 22 participants, Hernandez et al. [31]—45 patients, Schierz et al. [33]—65 patients, and Canning et al. [21]—73 patients. Condylographic examination is time-consuming but provides precise articulating parameters. It would be beneficial to examine more patients in each group for verification of the achieved results. Additionally, further studies will enable the provision of a precise correction for SCI according to the ANB angle.

In summary, prosthetic restorations should provide both missing structures and functions. The use of condylography enables the precise evaluation of condyle movement and thus reproduces and reconstructs the occlusal relations. Due to the similar values of SCI and TCI, standard parameters can be used in different skeletal classes. However, due to the wide individual variation in SCI and TCI values, it is advisable to use individual measurable parameters of mandibular movements, especially in complex prosthetic reconstructions. There is a linear statistic relationship between the SCI and the ANB angle, thus, in significant skeletal disproportions, the corrected articulating parameters should be applied. Additionally, the use of individualized values is especially important for patients with lowered adaptation capacities.

## Figures and Tables

**Table 1:** Sagittal condylar inclination (SCI) of the left temporomandibular joint (TMJ).

Class	N	Min	Q1	Median	Average	Q3	Max	SD	SE
All	75	32	43.0	50.0	49.2	55.5	70	8.5	0.98
I	23	32	45.8	51.5	51.0	55.8	70	7.9	1.65
II	29	39	47.0	52.0	51.8	56.0	70	7.6	1.41
III	23	32	37.2	42.5	43.6	49.5	61	8.1	1.69

N, number of patients in the group; Min, minimal recorded value of the measure; Q1, first quartile of the recorded value of the measure; Median, median recorded value of the measure; Average, average recorded value of the measure; Q3, third quartile of the recorded value of the measure; Max, maximum recorded value of the measure; SD, standard deviation of the recorded value of the measure; SE, standard error of the average recorded value of the measure.

**Table 2:** Sagittal condylar inclination (SCI) of the right TMJ.

Class	N	Min	Q1	Median	Average	Q3	Max	SD	SE
All	75	24	44.0	50	49.7	55.0	70	9.3	1.07
I	23	40	49.2	52	52.2	55.8	70	6.7	1.40
II	29	28	46.8	52	51.8	55.2	70	9.2	1.71
III	23	24	38.0	41	44.2	50.0	64	9.7	2.02

N, number of patients in the group; Min, minimal recorded value of the measure; Q1, first quartile of the recorded value of the measure; Median, median recorded value of the measure; Average, average recorded value of the measure; Q3, third quartile of the recorded value of the measure; Max, maximum recorded value of the measure; SD, standard deviation of the recorded value of the measure; SE, standard error of the average recorded value of the measure.

**Table 3:** Transversal condylar inclination (TCI) of the left temporomandibular joint (TMJ).

Class	N	Min	Q1	Median	Average	Q3	Max	SD	SE
All	75	0	0.0	3.0	4.1	6.0	18	4.6	0.53
I	23	0	1.5	5.5	6.0	10.5	17	5.0	1.04
II	29	0	0.0	3.0	3.2	5.0	16	3.6	0.67
III	23	0	0.0	2.0	3.6	4.0	18	5.1	1.06

N, number of patients in the group; Min, minimal recorded value of the measure; Q1, first quartile of the recorded value of the measure; Median, median recorded value of the measure; Average, average recorded value of the measure; Q3, third quartile of the recorded value of the measure; Max, maximum recorded value of the measure; SD, standard deviation of the recorded value of the measure; SE, standard error of the average recorded value of the measure.

**Table 4:** Transversal condylar inclination (TCI) of the right TMJ.

Class	N	Min	Q1	Median	Average	Q3	Max	SD	SE
All	75	0	0	2	4.3	7	22	5.2	0.60
I	23	0	0	3	5.2	7	22	6.0	1.25
II	29	0	0	2	4.1	7	15	5.1	0.95
III	23	0	0	2	3.6	5	16	4.5	0.94

N, number of patients in the group; Min, minimal recorded value of the measure; Q1, first quartile of the recorded value of the measure; Median, median recorded value of the measure; Average, average recorded value of the measure; Q3, third quartile of the recorded value of the measure; Max, maximum recorded value of the measure; SD, standard deviation of the recorded value of the measure; SE, standard error of the average recorded value of the measure.

**Table 5:** The linear regression models.

	The Coefficient for the ANB Angle	The Average from the Model	The *p*-Value of Test F	R^2^
SCI Left	0.77	47.5	0.012	0.098
SCI Right	0.72	48.5	0.039	0.069

## Data Availability

The data presented in the study are available on reasonable request from authors of this article.
